# Supplementary material for: Invasomes and Nanostructured Lipid Carriers for Targeted Delivery of Ceftazidime Combined with N-Acetylcysteine: A Novel Approach to Treat Pseudomonas aeruginosa-Induced Keratitis
Source: Pharmaceutics. 2025 Sep 11;17(9):1184. doi: 10.3390/pharmaceutics17091184 (PMC12473240; doi:10.3390/pharmaceutics17091184)
Supplement: Supplementary file 1 [file pharmaceutics-17-01184-s001.zip › pharmaceutics-3837844-supplementary.pdf]

Article

Invasomes and Nanostructured Lipid Carriers for Targeted Delivery of Ceftazidime Combined with N-Acetylcysteine: A Novel Approach to Treat *Pseudomonas aeruginosa*-Induced Keratitis

Mina Josef [1], Menna M. Abdellatif [1,*], Rehab Abdelmonem [1,*], Mohamed A. El-Nabarawi [2], Mahmoud Teaima [2], Hadeer M. Bedair [3] and Alshaimaa Attia [1]

[1] Department of Industrial Pharmacy, College of Pharmaceutical Sciences and Drug Manufacturing, Misr University for Science and Technology, P.O. Box 77, Giza 12566, Egypt; mina.azmy@must.edu.eg (M.J.); elshimaa.mohamed@must.edu.eg (A.A.)
[2] Department of Pharmaceutics and Industrial Pharmacy, Faculty of Pharmacy, Cairo University, El-Kasr El-Aini Street, Cairo 11562, Egypt; mohamed.elnabarawi@pharma.cu.edu.eg (M.A.E.-N.); mahmoud.teaima@pharma.cu.edu.eg (M.T.)
[3] Department of Microbiology and Immunology, College of Pharmaceutical Sciences and Drug Manufacturing, Misr University for Science and Technology, P.O. Box 77, Giza 12566, Egypt; hadeer.bedair@must.edu.eg
* Correspondence: menna.abdallatif@must.edu.eg (M.M.A.); rehab.abdelmonem@must.edu.eg (R.A.)

Supplementary Materials

Academic Editors: Jelena Filipović-Grčić and Taher Hatahet

Received: 8 August 2025
Revised: 4 September 2025
Accepted: 8 September 2025
Published: 11 September 2025

Citation: Josef, M.; Abdellatif, M.M.; Abdelmonem, R.; El-Nabarawi, M.A.; Teaima, M.; Bedair, H.M.; Attia, A. Invasomes and Nanostructured Lipid Carriers for Targeted Delivery of Ceftazidime Combined with N-Acetylcysteine: A Novel Approach to Treat *Pseudomonas aeruginosa*-Induced Keratitis. *Pharmaceutics* **2025**, *17*, 1184. https://doi.org/10.3390/pharmaceutics17091184

Copyright: © 2025 by the authors. Licensee MDPI, Basel, Switzerland. This article is an open access article distributed under the terms and conditions of the Creative Commons Attribution (CC BY) license (https://creativecommons.org/licenses/by/4.0/).

Table S1. Experimental runs, factors, and computed response of the 2^3 factorial design of CTZ-loaded INVs.

Formulation Code	Limonene concentration (%) (X_1)	Lipid amount (%) (X_2)	Sonication time (min) (X_3)	EE (%) (Y_1)	PS (nm) (Y_2)	PDI (Y_3)	ZP (mV) (Y_4)	Q8 (%) (Y_5)
F1	0.30	50	10	67.55±1.6	84.12±3.3	0.423±0.002	27.0±0.21	75.09±1.6
F2	0.30	50	20	64.31±0.91	164.90±3.5	0.421±0.005	23.1±0.77	92.21±2.7
F3	0.10	50	20	67.55±1.3	91.86±0.57	0.520±0.005	29.0±0.24	83.15±2.6
F4	0.30	150	10	79.88±0.39	238.50±4.2	0.447±0.001	36.4±0.07	66.99±0.36
F5	0.10	50	10	79.23±0.51	43.50±0.53	0.598±0.018	31.8±1.3	74.36±2.2
F6	0.20	100	15	77.30±0.14	155.0±2.2	0.529±0.013	32.4±0.09	74.16±1.9
F7	0.30	150	20	73.07±1.1	338.40±3.5	0.557±0.011	40.1±0.56	77.49±1.96
F8	0.10	150	20	85.08±1.4	149.4±2.1	0.471±0.013	33.3±0.20	79.60±0.85
F9	0.10	150	10	87.34±0.52	110.0±0.35	0.420±0.010	33.7±0.35	63.95±1.1
F10	0.20	100	15	77.50±0.42	153.0±0.42	0.522±0.019	32.23±0.08	74.22±0.9
F11	0.20	100	15	77.00±0.70	153.50±2.8	0.530±0.012	32.11±0.01	74.09±0.2
F12	0.20	100	15	77.25±0.14	154.0±1.6	0.520±0.013	32.09±0.09	74.43±0.17

Abbreviations: EE%, entrapment efficiency percentage; CTZ, ceftazidime; PS, particle size; PDI, polydispersity index; and Q8%, cumulative percent of drug permeated after 8 h.

Table S2. Experimental runs, studied factors, and assessed response of the 2^3 factorial design of CTZ-loaded NLCs.

Formulation Code	Lipid concentration (X_1) (%)	Solid lipid percent (X_2) (%)	Oil type (X_3)	EE (Y_1) (%)	PS (Y_2) (nm)	PDI (Y_2)	ZP (Y_4) (mV)	Q8 (Y_5) (%)
F1	3	90	Labrasol	85.30±1.2	262.4±7.35	0.316±0.012	29.27±0.98	69.42±0.57
F2	2.5	75	Labrasol	85.69±0.56	194.6±0.77	0.334±0.007	21.55±0.67	66.56±1.3
F3	2	90	Labrafac	82.17±0.91	195.7±3.18	0.511±0.022	18.94±0.55	77.59±1.7
F4	3	90	Labrafac	82.04±1.0	211.69±5.87	0.407±0.18	22.00±0.49	79.52±1.9
F5	2.5	75	Labrasol	85.69±0.78	195.2±8.27	0.34±0.011	21.55±0.061	66.56±1.3
F6	2	90	Labrasol	85.54±2.1	206.9±4.38	0.287±0.013	20.00±0.71	72.00±1.1
F7	3	60	Labrafac	84.34±1.4	210.7±0.42	0.379±0.009	18.20±0.82	69.74±1.2
F8	2	60	Labrafac	85.12±1.6	207.9±5.09	0.407±0.017	27.10±0.64	64.80±1.3
F9	2.5	75	Labrafac	83.42±2.1	191.2±4.94	0.441±0.012	21.38±0.42	70.42±2.1
F10	2	60	Labrasol	86.39±0.8	138.7±4.45	0.413±0.016	19.09±0.80	63.13±1.4
F11	3	60	Labrasol	84.40±1.6	179.5±2.75	0.378±0.006	17.22±1.6	61.68±0.8
F12	2.5	75	Labrafac	83.60±2.2	198.83±5.39	0.427±0.021	22.07±1.3	70.42±1.1

Abbreviations: EE%, entrapment efficiency percentage; CTZ, ceftazidime; PS, particle size; PDI, polydispersity index; and Q8%, cumulative percent of drug permeated after 8 h.
